# Evaluation of inter- and intra-fraction 6D motion for stereotactic body radiation therapy of spinal metastases: influence of treatment time

**DOI:** 10.1186/s13014-021-01892-5

**Published:** 2021-08-30

**Authors:** Ahmed Hadj Henni, David Gensanne, Maximilien Roge, Chantal Hanzen, Guillaume Bulot, Elyse Colard, Sebastien Thureau

**Affiliations:** Centre Henri Becquerel, 1 Rue d’Amiens, 76000 Rouen, France

**Keywords:** SBRT, CBCT, Spinal, Inter-fraction, Intra-fraction, Treatment time

## Abstract

**Background:**

The objective of this study was to analyze the amplitude of translational and rotational movements occurring during stereotactic body radiotherapy (SBRT) of spinal metastases in two different positioning devices. The relevance of intra-fractional imaging and the influence of treatment time were evaluated.

**Methods:**

*Twenty* patients were treated in the supine position either (1) on a body vacuum cushion with arms raised and resting on a clegecel or (2) on an integrated SBRT solution consisting of a SBRT table top, an Orfit™ AIO system, and a vacuum cushion. Alignments between the cone beam computed tomography (CBCT) and the planning computed tomography allowed corrections of inter- and intra-fraction positional shifts using a 6D table. The absolute values of the translational and rotational setup errors obtained for 329 CBCT were recorded. The translational 3D vector, the maximum angle, and the characteristic times of the treatment fractions were calculated.

**Results:**

An improvement in the mean (SD) inter-fraction 3D vector (mm) from 7.8 (5.9) to 5.9 (3.8) was obtained by changing the fixation devices from (1) to (2) (*p* < 0.038). The maximum angles were less than 2° for a total of 87% for (1) and 96% for (2). The mean (SD) of the intra-fraction 3D vectors (mm) was lower for the new 1.1 (0.8) positioning fixation (2) compared to the old one (1) 1.7 (1.7) (*p* = 0.004). The angular corrections applied in the intra-fraction were on average very low (0.4°) and similar between the two systems. A strong correlation was found between the 3D displacement vector and the fraction time for (1) and (2) with regression coefficients of 0.408 (0.262–0.555, 95% CI) and 0.069 (0.010–0.128, 95% CI), respectively. An accuracy of 1 mm would require intra-fraction imaging every 5 min for both systems. If the expected accuracy was 2 mm, then only system (2) could avoid intra-fractional imaging.

**Conclusions:**

This study allowed us to evaluate setup errors of two immobilization devices for spine SBRT. The association of inter- and intra-fraction imaging with 6D repositioning of a patient is inevitable. The correlation between treatment time and corrections to be applied encourages us to move toward imaging modalities which allow a reduction in fraction time.

## Background

Stereotactic *body* radiotherapy (SBRT) is a technique based on the concept of image-guided radiation therapy (IGRT) with very high precision that delivers high doses with a strong gradient associated with reduced margins. The objective is to obtain a high biological effective dose in a limited number of fractions. Stereotactic radiotherapy of spinal metastases can achieve (depending on the number of fractions and selected dose) local control at 1 year estimated to be between 80.6 and 92.7% according to a recent review of the literature including data from 38 studies [1].

The particular difficulty in this location is the millimetric proximity of the planning target volume (PTV) to nerve structures (spinal cord, cauda equina). Large dose gradients in the direction of these structures imply that greater precision must be guaranteed at all stages of patient management. A lack of precision can lead to serious side effects such as radiation myelopathy [2]. Spinal metastases SBRT fundamentally requires the use of accurate multimodal imaging [3], a ballistic using intensity modulation that can achieve a high degree of conformity in the irradiation of concave targets, and an image-guidance strategy associated with an immobilization system guaranteeing the accuracy of radiation delivery.

At the implementation of the technique in our center in 2018, several cone beam computed tomography (CBCT) scans were performed before and during treatment associated with a treatment table allowing submillimeter movements with six degrees of freedom. An initial vacuum cushion device was used (Pos_Old) and replaced in July 2019 with an integrated SBRT solution (Pos_New). This change was made to homogenize our immobilization practices to increase the comfort, accuracy and reproducibility of patient positioning. CBCT Imaging performed before and during treatment was acquired to minimize positioning errors.

The objective of this study was to quantify the amplitude of translational (vertical, longitudinal, and lateral) and rotational (pitch, yaw, and roll) movements in spinal metastasis SBRT using inter- and intra-fractional CBCT imaging. The relevance of intra-fraction imaging, which increases the time of treatment fraction, and the influence of this parameter on the offset errors were evaluated. This study also allowed the evaluation of two methods of immobilization, one based on the use of a half-body vacuum bag widely used in radiotherapy centers, and the other on a commercial solution completely integrated and indexed to the table, *which is not yet* studied in the literature to our knowledge. As our old positioning system (Pos_Old) was widely used for other treatment sites (e.g. lung) prior to the implementation of spinal SBRT, the radiotherapy technologists were therefore used to this type of positioning system. For the new device (Pos_New), the radiotherapy technologists received training from the vendor before use. It was also used for other treament sites. Therefore, the results have not been influenced by the users’ experience.

These results were compared with those of other studies to evaluate our practices and our level of positioning accuracy as recommended by learned societies such as the UK consortium [4].

## Methods

Our retrospective study received the institutional consent necessary for its realization. The clinical data analyzed were from a cohort of 20 patients treated at our center with stereotactic radiotherapy for one or more spinal lesions. The period covered was from February 2018 to September 2020.

### Patients and dosimetric planning

**Fig. 1:**
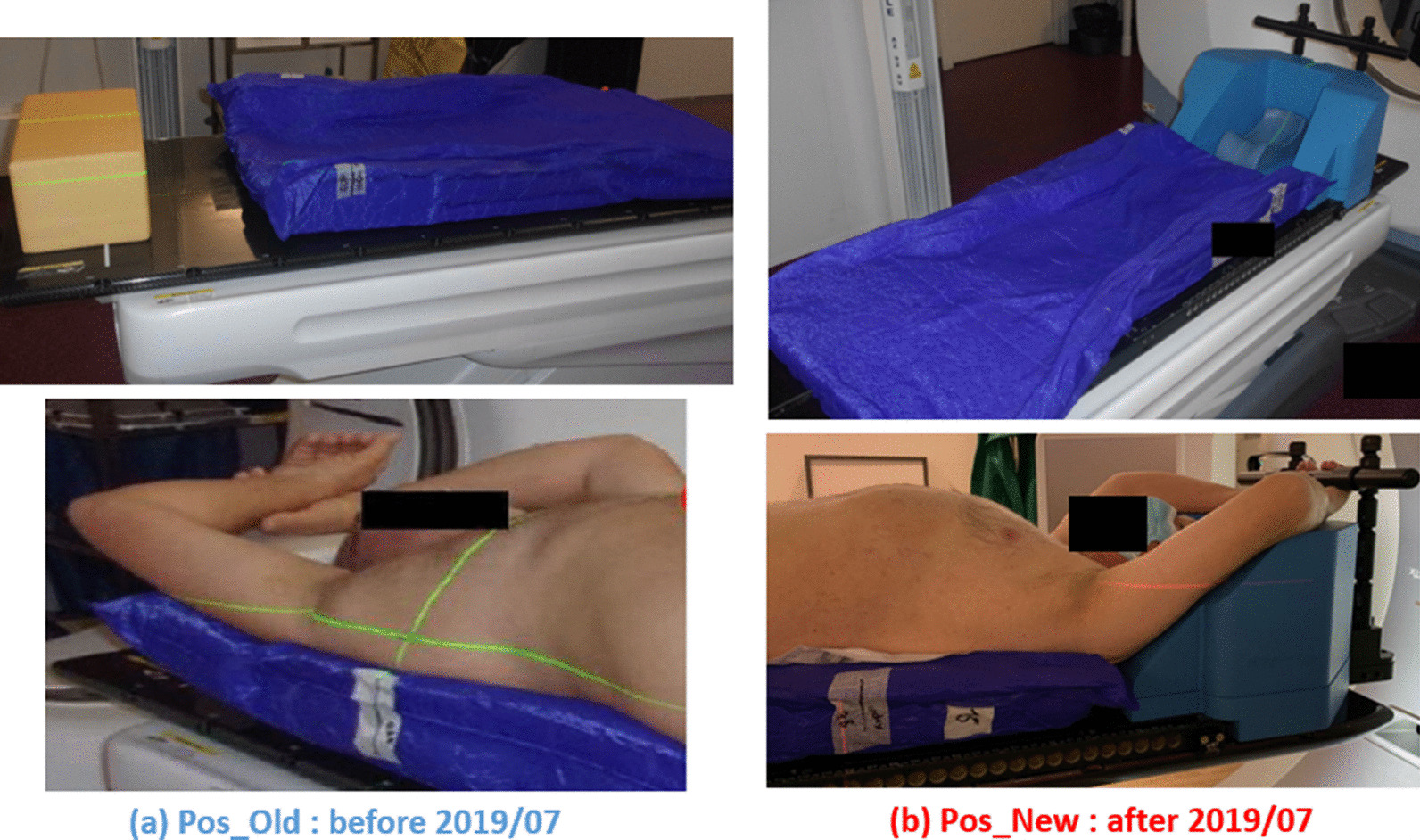
Patient positioning systems: **a** vacuum cushion, arms raised and resting on a clegecel and **b** AIO Orfit™ system and vacuum cushion

All 20 patients were in the supine position. Ten were placed in a half-body vacuum bag (VacQfix™; Qfix, Avondale, PA, USA) with their arms raised and resting on a clegecel (Pos_Old, Fig. 1a). The remaining ten were placed on an integrated SBRT solution consisting of an SBRT tabletop, an Orfit™ AIO system (Orfit Industries NV, Wijnegem, Belgium), and half-body vacuum bag (Pos_New, Fig. 1b).

CT imaging was acquired with a slice thickness of 1.25 mm [3] centered on the secondary bone lesion. Spinal MRI in the treatment position was also performed according to a standardized protocol combining 3D T2 and T1 sequences with gadolinium injection with a 1 mm isotropic voxel size.

The gross target volume (GTV) was delineated by experienced radiation oncologists after registration of the different imaging modalities (CT, MRI and PET CT if available). The clinical target volume (CTV) was adapted according to the anatomical vertebral bone segment invaded by the GTV as specified in international recommendations [5–7]. A margin of 2 mm was applied from the CTV to obtain the PTV. This margin was reduced to 0 mm, closed to the spinal cord.

All treatments were performed with volumetric modulated arc therapy (VMAT) according to our dosimetric protocol based on three coplanar arcs in 6MV photons at 600 MU/min and collimator angles of 45°, 315° and 95°. All treatment plans were calculated using the same planning system (Eclipse AAA 13.6.23, 0.125 cm grid size; Varian Medical Systems).

### Irradiation and imaging strategy during treatment

All patients were planned on a Truebeam Stx (Varian Medical Systems, Palo Alto, CA, USA) dedicated to stereotactic treatment and equipped with an MLC 120HD (high definition), an on-board imager (OBI) and a PerfectPitch table with six degrees of freedom. The quality controls performed on our linear accelerator were those recommended by learned societies with tolerance thresholds adapted to the level of requirements expected in stereotactic treatment [3].

To ensure optimal patient positioning, a pre-treatment CBCT (CBCTpTT) and CBCT between each of the three arcs (CBCT12 and CBCT23) were acquired corresponding to our intra-fraction imaging (CBCT12 + 23). CBCT scans were acquired with a gantry rotation of 360° (120 kV, 80 mA) with a slice spacing of 1.98 mm and pixel resolution of 0.91 mm and registered with a planning CT with a slice spacing of 1.25 mm and pixel resolution of 0.94 mm.

A senior radiation oncologist was present during treatment. The standardized approach was to first perform a manual bone registration on a large region of interest to avoid any possibility of vertebral error and then to automatically perform a registration on the volume of interest. Online automatic registration between the CBCT and planning CT was verified by a physician and manually adjusted when it was necessary and systematically applied using the 6D treatment table (accuracy of 0.1 mm/0.1°).

### Analysis methodology

Positional errors were collected for each CBCT via the Aria Offline review module (ARIA 13.6; Varian Medical System, Palo Alto, CA) for the three translational (mm) lateral (X, LR), vertical (Y, AP), and longitudinal (Z, CC) and rotational (°) pitch (R_x_), yaw (R_y_) and roll (R_z_) motions. The absolute values of the mean, standard deviation, and maximum value of each motion were calculated. A 3D displacement vector (3D Vect = $\sqrt {{X^2} + {Y^2} + {Z^2}}$) and the maximum angle of the rotations (Max Angle = Max(R_X_, R_y_, R_z_)) were deduced.

Typical treatment fraction times were also *calculated* using the Aria Offline Review tool. The total treatment time of a session was counted from the beginning of the CBCTpTT acquisition to the completion of the last arc (arc 3). The first characteristic intra-fraction time was calculated from the start of the CBCTpTT acquisition to the start of the CBCT12 acquisition. The second intra-fraction time recorded corresponded to the time between the start of CBCT12 and the start of CBCT23. These two times were related to the offsets given by CBCT12 and CBCT23.

Two groups of data were compared corresponding to the two immobilization systems used. The significance of this comparison was assessed using a non-parametric Mann–Whitney test. The results were associated with a *p* value with a threshold value of 0.05 below which the difference was considered significant. The evidence of a link between the treatment time and 3D translational deviation (3D Vect) was based on the results of Pearson (r, *p* value) and Spearman (rho, *p* value) correlation coefficients. All statistical tests were performed using the XLSTAT statistical software (version 2020.5).

## Results

### Overview of a spine SBRT treatment

In our cohort of 20 patients, 22 vertebrae were treated with stereotactic radiotherapy. Of these patients, 35% (n = 7) were men and 65% (n = 13) were women with a median age of 66 years (range, 48–76 years). Breast cancer was the most common primary cancer (50%) followed by prostate cancer (25%), lung cancer (20%) and small bowel cancer (5%).

The most frequent histological types were adenocarcinoma (55%) for bronchial tumors and infiltrating ductal carcinomas (35%) for breast tumors. One patient (5%) had an infiltrating lobular carcinoma and one patient had a neuroendocrine tumor (5%).

Vertebral lesions were treated in thoracic spine cases (55%), lumbar spine cases (41%), and sacrum cases (4%). The series included only one adjuvant situation on a single vertebra (postoperative). All patients received treatment at a dose of 35 Gy in 5 fractions of 7 Gy, three times a week, on an average GTV of 9.96 cm^3^ and an average CTV of 32.35 cm^3^. Patient and treatment characteristics are summarized in Table 1.

**Table 1:** Patients and treatment characteristics

Patients (n = 20)	
Sex	
Men	35% (n = 7)
Women	65% (n = 13)
Median age (years), (range)	66 (48–76)
Primary cancer	
Breast	50% (n = 10)
Prostate	25% (n = 5)
Lung	20% (n = 4)
Small bowel	5% (n = 1)
Histological types	
Adenocarcinoma	55% (n = 11)
Infiltrating ductal carcinoma	35% (n = 7)
Infiltrating lobular carcinoma	5% (n = 1)
Neuro-endocrine tumor	5% (n = 1)
Vertebral lesions	22
Vertebrae treated in post-operative situation	1
Location of spinal lesions	
Thoracic spine	55% (n = 12)
Lumbar spine	41% (n = 9)
Sacrum	4% (n = 1)
Treatment	
Mean GTV (cm^3^), (range)	9.96 (0.40–42.30)
Mean CTV (cm^3^), (range)	32.35 (8.74–99.50)
Prescribed dose	
5 × 7 Gy	100% (n = 20)

A total of 110 treatment fractions and 329 CBCT scans were retrospectively analyzed allowing us to evaluate our level of accuracy in the inter-fraction (110 CBCTpTT) and intra-fraction (110 CBCT12 and 109 CBCT23) for two different immobilization systems (179 Pos_Old and 150 Pos_New).

The mean (SD) characteristic data of the spinal stereotactic treatment fraction at our center were *calculated.* The number of MUs delivered per arc was 837 (198) MUs. When the old positioning device (Pos_Old) was used, the total treatment time (Imaging + irradiation) was 20.4 (3.7) min versus 17.1 (4.2) min for the new system (Pos_New) (*p* < 0.0001). The beam-on time (arc1 + arc2 + arc3) was 4.4 (0.8) min.

### Inter and intra-fraction results

The mean absolute(SD) inter-fraction setup errors for six degrees of freedom are listed in Table 2. The results for the 3D translation vector and maximum rotation angle are presented in Table 3.

**Table 2:** Summary of translational and rotational deviation of each positioning system for each type of CBCT

CBCT	Abosolute translational shifts (mm) Mean (SD, Max)			Absolute rotational shifts (°) Mean (SD, Max)		
	X (Lat.)	Y (Vert.)	Z (Long.)	Rx (Pitch)	Ry (Yaw)	Rz (Roll)
Pos_Old (179)						
CBCTpTT (60)	4.0 (3.7, 15.9)	2.4 (2.2, 9.5)	5.0 (5.5, 26.4)	0.8 (0.6, 2.2)	1.0 (0.7, 2.7)	0.9 (0.7, 2.7)
CBCT12 + 23 (119)	0.9 (0.9,5.2)	0.6 (0.7, 4.1)	0.9 (1.5, 9.6)	0.2 (0.3, 2.5)	0.2 (0.3, 1.8)	0.2 (0.3, 2.2)
Pos_New (150)						
CBCTpTT (50)	3.1 (3.7, 22.0)	1.6 (1.3, 6.2)	3.6 (3.1, 10.9)	0.7 (0.6, 2.3)	0.9 (0.7, 2.0)	0.9 (0.7, 2.4)
CBCT12 + 23 (100)	0.5 (0.5, 2.7)	0.6 (0.7, 4.7)	0.5 (0.5, 2.9)	0.2 (0.3, 1.3)	0.2 (0.3, 1.9)	0.2 (0.3, 1.9)

**Table 3.** 3D vector (mm) and max angle (°) calculated for each positioning system for each CBCT type

CBCT	3D vect (mm) Mean (SD, Max)	Max angle (°) Mean (SD, Max)
	3D vect = $\sqrt {X^{2} + { }Y^{2} { } + { }Z^{2} }$	Max angle = Max(R_X_; R_y_; R_z_)
Pos_Old (179)		
CBCTpTT (60)	7.8 (5.9, 28.1)	1.5 (0.6, 2.7)
CBCT12 + 23 (119)	1.7 (1.7, 9.7)	0.4 (0.4, 2.5)
Pos_New (150)		
CBCTpTT (50)	5.9 (3.8, 2.2)	1.4 (0.5, 2.4)
CBCT12 + 23 (100)	1.1 (0.8, 5.7)	0.4 (0.3, 1.9)

### Inter-fraction results

The absolute offsets from the CBCTpTT allowed us to evaluate the reproducibility of patient positioning in each immobilization devices. The means(SD) of the 3D vectors for the two immobilization systems Pos_Old and Pos_New were respectively 7.8 (5.9) mm and 5.9 (3.8) mm with a significant difference (*p* = 0.038). However, the difference observed between the means (SD) of the maximum angles, 1.5 (0.6)° for Pos_Old and 1.4 (0.5)° for Pos_New, was not statistically significant (*p* = 0.10). The results obtained for each of the translations and each of the rotations are detailed in the CBCTpTT section of Table 2.

**Fig. 2:**
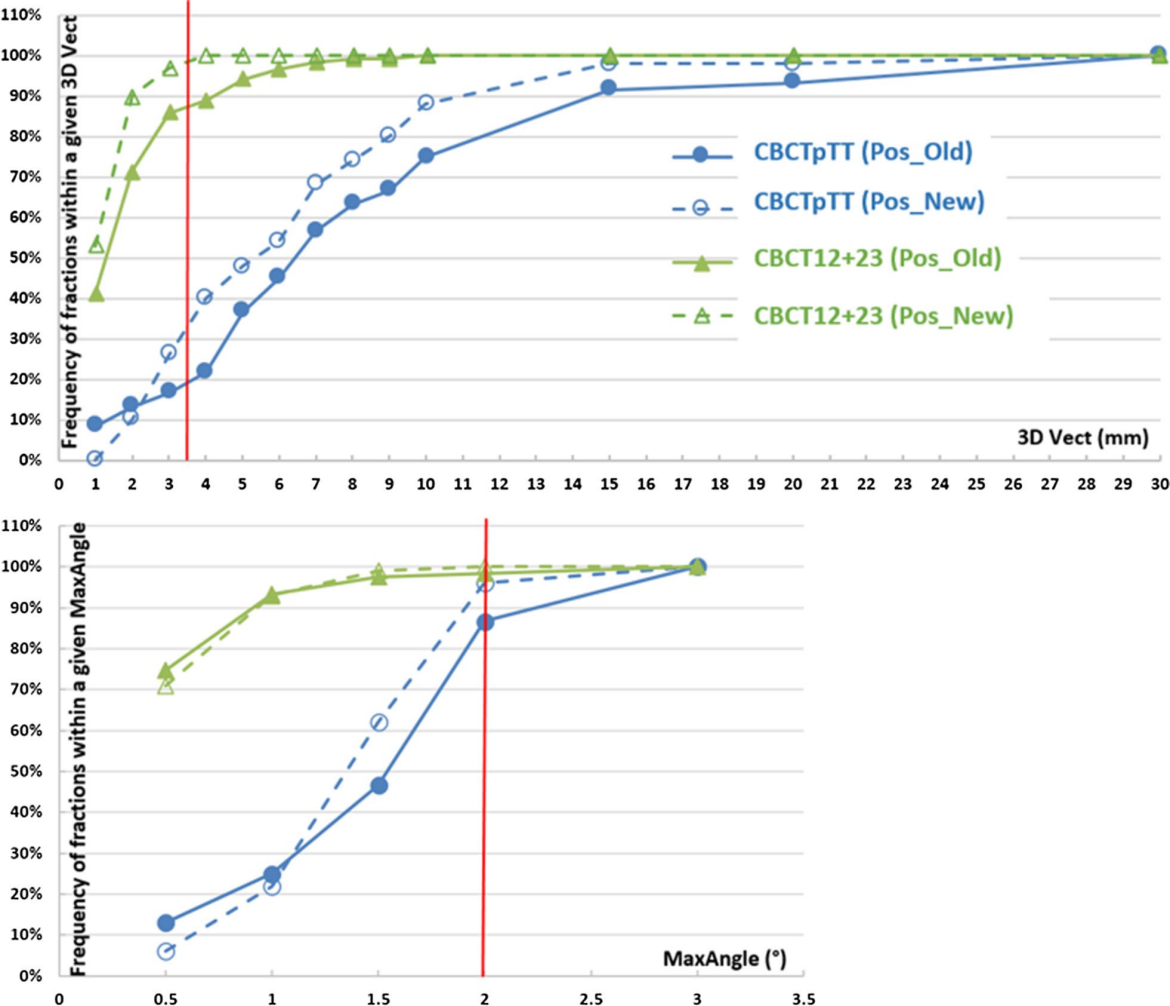
Proportion (%) of treatment fractions setup errors within given tolerance, translational (3D Vect on top) and rotational (MaxAngle at the bottom), measured on pre-treatment (CBCTpTT) and intra fractions (CBCT12 + 23) CBCTs. The thresholds denoted be a red line represent a 3D vector = 3.5 mm corresponding to a setup errors of 2 mm in the 3 directions and a maximum rotation angle of 2°

The CBCTpTT(Pos_Old) and CBCTpTT(Pos_New) curves in Fig. 2 represent the proportion (%) of treatment fractions setup errors within given tolerance, translational (3D Vect on top) and rotational (MaxAngle at the bottom), measured on pre-treatment CBCTs. For translational movements the comparaison of the two immobilization systems was performed using a 3D tolerance equal to 3.5 mm corresponding to setup errors of 2 mm in the three axes. In terms of rotation, a value of 2° represented the threshold beyond which the dosimetric impact was considered significant [8].

### Intra-fraction results

The results (mean, SD, and maximum value) for each translational and rotational intra-fraction offset applied (CBCT12 + CBCT23) for both immobilization systems are summarized in Table 2.

The means (SD) of the 3D vectors were significantly lower for the new fixation device 1.1 (0.8) mm compared to the old positioning system 1.7 (1.7) mm (*p* = 0.004) (Table 3). Corresponding to our tolerance of 3.5 mm, 87.5% and 98.5% of the intra-fraction CBCT offsets for Pos_Old and Pos_New respectively were below this threshold.

No significant difference (*p* = 0.9) was observed in the mean (SD) of the maximum angle (°) applied during the treatment fraction between Pos_Old 0.4 (0.4)° and Pos_New 0.4 (0.3)°. Although these results showed a relatively small angular correction in the intra-fraction, the maximum value of this angle was 2.5° for Pos_Old but remained below 2° for Pos_New (1.9°). In terms of frequency, Fig. 2 shows a similar behavior of the two positioning systems; for example, 98% and 100% of the maximum rotational errors were less than 2° for Pos_Old and Pos_New respectively, showing identical accuracy for the 2 patient immobilization strategies.

### Treatment time and intra-fraction translational movements

**Fig. 3:**
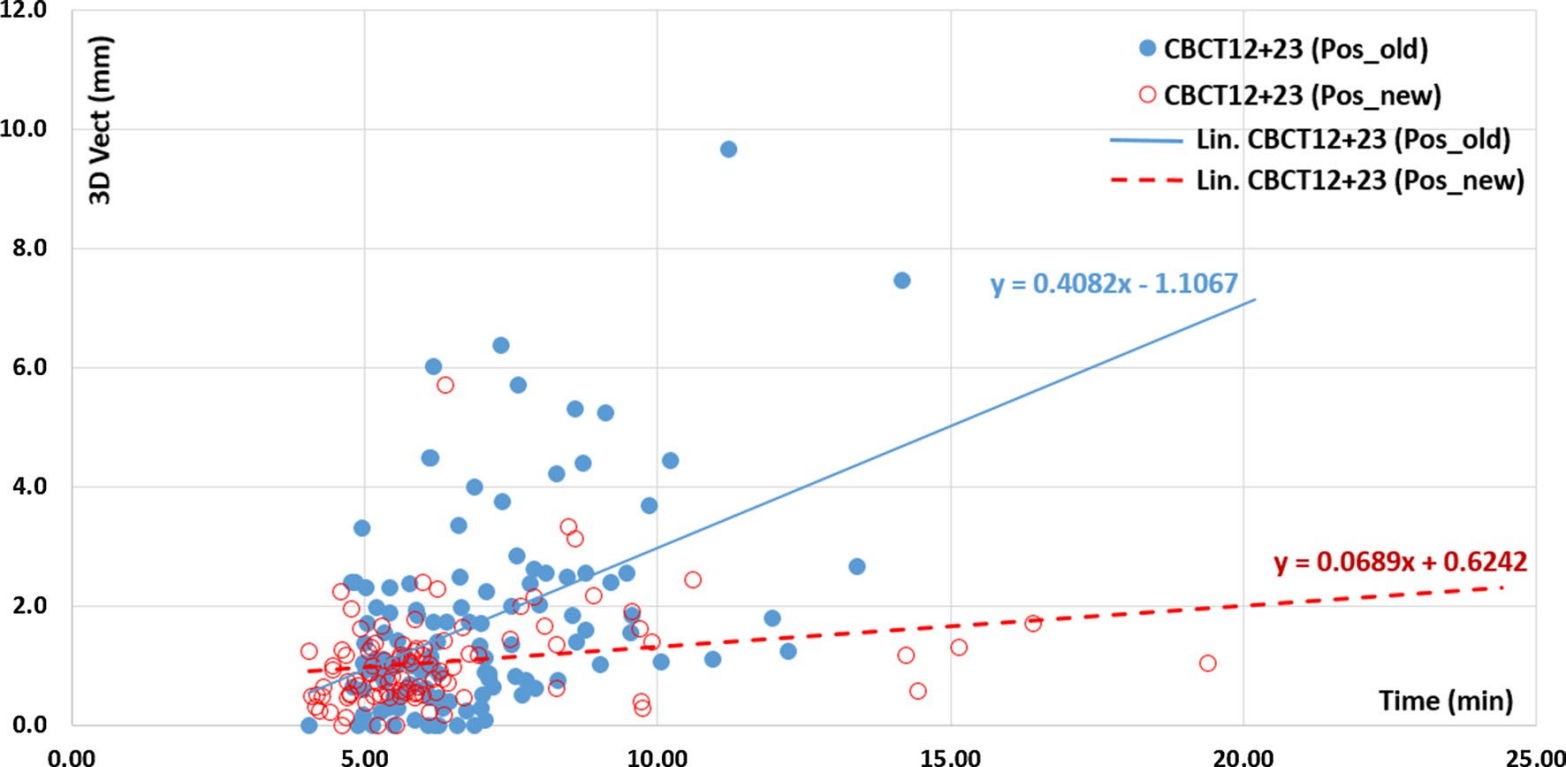
Relationship between intra-fraction 3D Vector (CBCT12 + 23) motion (mm) for Pos_Old (solid blue dots) and Pos_New (empty red dots) as a function of time (min) measured either between CBCTpTT start and CBCT12 start or between CBCT12 start and CBCT23 start. The linear fits are shown with their corresponding equations

Figure 3 shows the relationship between the 3D intra-fraction vector recorded between each arc and the treatment time. The latter represents the interval between either the start of CBCTpTT and the start of CBCT12 or between the start of CBCT12 and the start of CBCT23. These two durations correspond to intra-fraction times.

Our statistical tests, Pearson correlation coefficient (r, *p* value) and Spearman rank correlation coefficient (rho, *p* value), showed an increasing linear correlation between the 3D displacement vectors and time for the old positioning device Pos_Old (r = 0. 454, *p* < 0.0001) and (rho = 0.391, *p* < 0.0001), and the new positioning device Pos_New (r = 0.228, *p* < 0.021) and (rho = 0.383, *p* < 0.0001). Linear fits for Pos_Old and Pos_New are also presented in Fig. 3, with regression coefficients of 0.408 (0.262–0.555, 95% CI) and 0.069 (0.010–0.128, 95% CI), respectively. An estimate of the times by solving the two equations that involved exceeding our 3D threshold by 3.5 mm yielded 11.28 min for the old immobilization system and a time outside the characteristics of our spine stereotactic treatment fractions (41.7 min) for the new one. However, an expected accuracy of 1 mm in all directions (Vect 3D = 1.7 mm) would require intra-fractional imaging approximately every 5 min for the Pos_Old (5.2 min) and Pos_New (5.4 min) fixation devices.

## Discussion

The analysis of CBCT data before the beginning of the treatment fraction (CBCTpTT) and during (CBCT12 + 23) allowed us to evaluate our patient positioning practices and, consequently, determine if our margins were sufficient. This approach is strongly recommended by learned societies [4]. In fact, for spinal SBRT in France in 2016 [9], 71% used imaging during treatment.

Our results and a literature review on the subject led us to make improvements in the management of our patients treated for spinal metastasis with stereotactic body radiation therapy.

The comparison between the two positioning systems confirms our choice in favor of the new immobilization device. The inter-fraction positioning (CBCTpTT) was improved by the change from Pos_Old to Pos_New, both in translation and rotation; however, only 19.5% and 33%, respectively, of the 3D translational motions were less than a 3D threshold of 3.5 mm. These offsets found, and applied, during initial patient positioning (CBCTpTT) are relatively large regardless of the immobilization devices (see Tables 2, 3). To take into account this result an additional verification imaging was added just prior to treatment. This verification CBCT allows to assess the residual setup error after the initial image registration. This methodology is similar to the practice of 76% of the centers in France [9] and has also been reported in international literature [10, 11].

Wang et al. [8] considered that a loss of target volume coverage greater than 5% and an increase greater than 25% in the maximum dose to the OARs leads to significant dosimetric effects. Their goal, based on their study conducted on patients and phantoms, was to achieve a positioning accuracy for treatment ≤ 1 mm in translation and ≤ 2° in rotation. For GuckenBerger et al. [12], the dosimetric impact on the spinal cord was acceptable for maximum errors ≤ 1 mm and ≤ 3.5° on average.

In terms of frequency, 87% and 96% of rotations were less than 2° for the two immobilization devices Pos_Old and Pos_New respectively, with maximum values not exceeding 3°. Nevertheless, to guarantee the optimal accuracy, achieved by the new positioning system, we will continue to use accelerators equipped with a 6D table. According to a survey by Pougnet et al. [8], 81% of French centers use a 6D table to correct their positioning errors.

Concerning intra-fraction imaging, a review of the literature allowed us to compare our two types of fixation devices to the results reported by different studies [10, 11, 13, 14]. To evaluate our level of intra-fraction accuracy, we based the comparison on the standard deviations of the positioning errors, which are common to all the articles cited (Table 4).

**Table 4:** Comparison of standard deviations (SD) in mm of intra-fraction positioning errors found in the literature as well as the Pos_Old and Pos_New positioning systems

Authors	Positioning systems	SD (mm) for intra-fraction CBCT
Hyde et al	Elekta BodyFIX system	LR = 0.6; CC = 0.5 and AP = 0.5
Our center	Pos_New	LR = 0.5; CC = 0.5 and AP = 0.7
Dahele et al	Posirest	LR = 0.9; CC = 0.6 and AP = 0.7
Li et al	Elekta BodyFIX system	LR = 0.9, CC = 0.7 and AP = 0.9
Li et al	Evacuated cushion	LR = 1.3; CC = 1.2 and AP = 1.0
Li et al	Thermoplastic S-frame	LR = 1.3, CC = 0.9, and AP = 1.1
Foster et al	Evacuated cushion	LR = 1.05; CC = 1.23 and AP = 1.04
Our center	Pos_Old	LR = 0,9; CC = 1,5 and AP = 0,7

The Pos_Old system had the highest SD in the cranio-caudal (CC) direction (1.5 mm) among the positioning devices described in Table 4. However, the results obtained by our commercial Pos_New immobilization system were comparable to more rigid ones (BodyFIX, Medical Intelligence, Elekta, Schwabmunchen, Germany) when compared to Hyde et al. [11] and even slightly better when compared to Li et al. [10]. The advantage of the Pos_New system is that it is much less restrictive than a system using a polyethylene sheet under vacuum and is less time-consuming for pretreament setup. In addition, in the case of VMAT, the position of the arms along the body of the BodyFix device [10, 11] can restrict the number of radiation beam entries in order to reduce low dose to this organ. It should be noted that a simple Posirest [14] positioning system provided results close to those obtained by Hyde et al. [11] and our new Pos_New system. From this result, we postulate that arm immobilization is a determining factor for improving positioning quality. This hypothesis could explain why the old immobilization strategy based on the use of a vacuum cushion is inferior to that reported by Li et al. [10] and Foster et al. [13] with similar materials. Moreover, the standard deviation in the cranio-caudal direction 1.5 mm greater than the other two directions may confirm this hypothesis. Immobilization based on the use of a thermoformed mask for cervical locations [10] is classified at the same level of precision as a vacuum cushion.

The intra-fraction accuracy of our Pos_New system in translation and rotation (98.5% of 3D Vect ≤ 3.5 mm and 100% of Max Angle ≤ 2°, Fig. 2) could allow us to dispense with CBCT between each arc for a gain in treatment time. However, the analysis of the maximum values (respectively for 3D Vect and Max Angle 5.7 mm and 1.9°) leads us to keep intra-fraction imaging. The imaging systems on Truebeam Stx (Varian Medical Systems, Palo Alto, CA, USA) have the capability to perform triggered imaging (depending on MU, time or degrees) during irradiation. However, this only allows visual verification by juxtaposing the target volume contour on the kV2D acquisition. Another possibility is to opt for non-embedded kV-2D systems whose image acquisition is fast and allow an automatic registration without interruption of the beam delivery. Chang et al. [15] for example validated phantoms and patients with the kV-2D Exactrac system from Brainlab (Brainlab AG, Munich, Germany) as an alternative to CBCT in spine SBRT. Oh et al. [16] and Wang et al. [17] conducted the same study for intracranial and head and neck stereotactic radiotherapy respectively, and validated this system. However, the authors pointed out that precautions must be taken if this type of imaging is used alone.

In contrast to the results presented by Dahele et al. [14] for a Posirest positioning system, we found a strong correlation between the magnitude of 3D translation errors and intra-fraction time. If the goal is to achieve an accuracy of ≤ 1 mm, imaging should be performed every 5 min regardless of the fixation devices studied. The rotational intra-fraction offsets were well below 1°. These results are consistent with those presented in 2009 by Ma et al. [18]. Ma et al. determined the time of realization of a control imaging to maintain an accuracy of ≤ 1 mm and ≤ 1° to be between 5 and 7 min. If the goal is a precision of 2 mm, then the new Pos_New system theoretically allows the discarding of intra-fraction imaging.

These results should encourage teams to focus on optimizing the workflow of their treatment fraction to minimize positioning errors and the loss of biological effectiveness highlighted by several publications [19, 20] beyond a treatment time of more than 30 min.

In a larger study aiming at calculating the margins to be used, it would be necessary to evaluate the inter and intra observer registration error as well as the impact of the CBCT image quality on the automatic image registration. This uncertainty is typically much larger [21–23] than the accuracy allowed by 6D table (0.1 mm/0.1°). We had a cohort of 20 patients for 22 vertebrae with a total of 329 CBCT analyzed (110 CBCTpTT + 219 CBCT12 + 23) that allowed us to obtain statistically significant conclusions. For comparison, Dahele et al. [14] used data from 249 intra-fraction CBCTs; Finnigan et al. [24] used 225 image registrations. Li et al. [10] used a total of 355 localizations, 333 verifications, and 248 mid- and 280 post-treatment CBCTs. Other studies have used larger statistics, Foster et al. [13] for example used SBRT data from 141 lung, 29 liver, 48 prostate and 45 spine tumors.

This study dealt mainly with lumbar and thoracic locations. Cervical lesions whose immobilization system was based on thermoformed masks were not included in the results. As other locations outside the vertebrae (scapula, humerus) where patient positioning can sometimes be complicated, even if the proximity of sensitive OARs is less important. Moreover, CBCT acquired during treatment does not provide the exact moment when a patient's movements occur. Studies that use real-time imaging to track patient motion during irradiation could answer this question more accurately [25]. The use of flattening filter free (FFF) beams is also a tool for improving the speed of delivery of radiation verified from doses per fraction of 4 Gy in X6FFF and 10 Gy in X10FFF while keeping dosimetric results equivalent to filtered beams [26–28].

## Conclusions

In summary, this study evaluated setup errors of two immobilization devices used in the management of spinal stereotactic body radiation therapy. Improvements were made that increased the level of treatment accuracy. The correlation between treatment time and intra-fraction motion leads to the use of faster imaging modalities reducing the treatment time per fraction.

## Data Availability

All data and materials have been presented in the manuscript.

## Abbreviations

SBRT: Stereotactic body radiation therapy
CT: Computed tomography
CBCT: Cone-beam computed tomography
MRI: Magnetic resonance imaging
PET: Positron emission tomography
GTV: Gross target volume
PTV: Planning target volume
CTV: Clinical target volume
VMAT: Volumetric modulated arc therapy
AAA: Analytical anisotropic algorithm
SD: Standard deviation
AP: Ant-post
CC: Cranio-caudal
LR: Left–right
